# Stayability in Simmental cattle as affected by muscularity and body condition score between calvings

**DOI:** 10.3389/fvets.2023.1141286

**Published:** 2023-03-24

**Authors:** Giovanni Buonaiuto, Nicolas Lopez-Villalobos, Angela Costa, Giovanni Niero, Lorenzo Degano, Ludovica Maria Eugenia Mammi, Damiano Cavallini, Alberto Palmonari, Andrea Formigoni, Giulio Visentin

**Affiliations:** ^1^Department of Veterinary Medical Science, Alma Mater Studiorum – University of Bologna, Bologna, Italy; ^2^School of Agriculture and Environment, Massey University, Palmerston North, New Zealand; ^3^Department of Agronomy, Food, Natural Resources, Animals and Environment, University of Padova, Padova, Italy; ^4^National Association of Italian Simmental Cattle Breeders (ANAPRI), Udine, Italy

**Keywords:** culling, dual-purpose cows, functional longevity, resilience, survival rate

## Abstract

The present study aimed to investigate the association between stayability (STAY) traits, muscularity, and body condition score (BCS) in the Italian Simmental dual-purpose cows. Data were collected from 2,656 cows linearly scored in their first lactation from 2002 to 2020 and reared in 324 herds. The binary trait STAY, which is the ability of a cow to stay in the herd, was obtained for each cow-lactation available up to parity 5 (from STAY1-2 to STAY4-5). Analysis of STAY was carried out using logistic regression, considering the fixed effect of energy corrected milk, conception rate, somatic cell score, and muscularity or BCS predicted at different time points. The herd of linear classification and residual error were the random effects. Primiparous cows with a medium BCS and muscularity in early lactation presented a more favorable STAY across life compared to thinner ones (*P* < 0.05). In fact, cows with an intermediate BCS/muscularity were more likely to stay in the herd after the third lactation (STAY3-4), compared to those presenting a lower BCS/muscularity (*P* < 0.01). However, cows whose muscularity was high were generally less likely to start the third lactation compared to the others. A potential explanation for this could be the willing to market cows with good conformation for meat purpose. Simmental is in fact a dual-purpose breed known for the good carcass yield and meat quality. This study demonstrates how muscularity and BCS available early in life can be associated with the ability of Simmental cows to stay in the herd.

## Introduction

Herd-testing for daily milk yield and composition is one of the major sources of information for the genetic evaluation of dairy cows and quantification of herd productivity and profitability (1). For several decades, in European and Northern American countries, dairy breeding objectives included mainly traits related to milk production (2). Although being one the key drivers of profitable dairy farming, the genetic improvement for such characteristics has led to deterioration of functional traits, such as longevity and fertility, due to the antagonistic genetic correlations existing among these traits (3–5). For these reasons, nowadays breeding objectives of dairy cattle include a plethora of economically relevant traits not necessarily directly related to milk productivity (2, 6). In dairy farming, high culling rates indicate poor animal welfare, suboptimal farming conditions, and inefficient use of animal resources, impairing the sustainability of the dairy sector (7). According to Allendorf and Wettemann (8), high replacement rates cause a decrease in herd productivity followed by augmented replacement costs (9). Indeed, in Holstein-Friesian cows, milk production per lactation is maximized at the third lactation (10) and cows usually finish paying back their initial rearing cost at the end of their second lactation (11). Thus, culling cows before that moment has a detrimental impact on farmers' profitability (12, 13). Moreover, low culling rates may also improve the environmental footprint of dairy farms because of the lower number of heifers required in the herd (14).

In dairy cows, productive lifetime is defined as the period during which the animal is in production in the herd (15). Instead, longevity can be described in different manner in dairy cattle: e.g., by means of age at last calving, number of lactations started or completed, number of days from first calving to culling, age at culling, and survival at various ages or parities. Longevity combines all the characteristics that are directly associated with a cow's ability to successfully stay and perform in the herd (16). For this reason, some authors (17–19) have opted for the term “stayability” (STAY). This trait can be considered somehow equivalent to longevity, but it is usually expressed in a binary trait where 1 and 0 indicate if the animal remains in the herd and produces up to a specific moment or not, respectively (20). Cow's STAY is a key component of profitability in dairy production, as long-living cows allow to achieve the same herd production with a lower replacement rate. This implies that replacement costs can be reduced and that surplus newborn calves, preferably crossbred, can enter the beef market (21).

Conformation traits, or type traits, have been used for indirect selection to improve productive life due to their correlation with survival (22). Although collection of such phenotypes is consuming in terms of time and labor, the main advantage of type traits is that they are available early in life (2), and, indeed, several authors reported correlation of some type traits with longevity in different dairy cow breeds. Jovanovac and RaguŽ (23) reported that udder and body conformation traits, as well as muscularity and size traits, could be used as predictors of STAY and longevity in Croatian Simmental dairy cows. Schneider et al. (24) reported that udder and feet and legs traits had a strong relationship with functional herd life in Quebec Holsteins. In addition, Imbayarwo-Chikosi et al. (25) reported that chest width and rump angle were strongly associated with the risk of culling in South African Holstein dairy cows. To the best of our knowledge, no studies have attempted to identify factors associated with STAY in Italian Simmental cows, whose breeding objectives include both dairy and beef attitudes. This would be important to inform farmers in optimizing management and culling decisions based on some conformation traits, recorded within the national recording scheme.

Therefore, the aim of the present study was to retrospectively explore the variability of STAY in Italian Simmental cows and quantify its association with muscularity and BCS.

## Materials and methods

### Database

The present study was conducted using data retrieved from the National Association of Italian Simmental Cattle Breeders (ANAPRI, Udine, Italy) database that were collected between January 2002 and December 2020. Data was recorded on 2656 Italian Simmental dual-purpose cows reared in 324 dairy herds located in Emilia Romagna region, in North-eastern Italy. This region has a large number of dairy farms [3,519; BDN-Anagrafe Zootecnica Nazionale, (26)] that greatly contribute to the regional economy [60% of the regional gross saleable production; ISTAT, (27)]. The majority of the farms involved in the present research were multi-breed, i.e., the 97% of farms reared both Simmental and Holstein cows. Out of these, in 15 farms the number of Simmental cows was equal or above 50; only 2 of them had more than 130 heads. Only cows which were linearly classified in their first lactation were considered in the present study.

Data provided by ANAPRI included information regarding the cows' lactations estimated by the Italian Breeders Association (AIA, Rome, Italy), namely whole lactation milk and solids yield, and test-day milk records with the daily milk yield, gross composition, and somatic cell count (SCC, cells/mL). Linear type traits scores, measured once in life (in primiparous cows) by trained personnel were also present.

### Phenotypes

#### Stayability

This trait was calculated in the lactation set, and it was defined for each cow-lactation up to the fifth, based on the presence or absence of the subsequent calving date (Table 1). Briefly, a STAY equal to 1 was assigned if a calving date was present after the previous lactation, otherwise STAY was considered equal to 0. The value was recursively set at 0 for all parities after the one incurring the culling date. This resulted in five different variables for each cow: STAY1-2, STAY2-3, STAY3-4, and STAY4-5.

**Table 1:** Definition and descriptive statistics of the stayability traits.

**Trait**	**Definition**	**Cows^a^**	**Rate^b^**
STAY1-2	Stayability as a primiparous cow: survived to 1st lactation = 1; departed during 1st lactation *=* 0.	2,656	0.98
STAY2-3	Stayability as a second-parity cow: survived to 2nd lactation *=* 1; Departed during 2nd lactation *=* 0.	2,603	0.70
STAY3-4	Stayability as a third-parity cow: Survived to 3rd lactation *=* 1; Departed during 3rd lactation *=* 0.	1,819	0.62
STAY4-5	Stayability as a fourth-parity cow: survived to 4th lactation *=* 1; Departed during 4th lactation *=* 0.	1,127	0.57

a Number of cows survived.

b Proportion of cows survived (STAY = 1).

#### Milk traits

The energy corrected milk (ECM) was obtained from the actual lactation data according to the formula proposed by Dairy Records Management Systems (28):


$$\begin{array}{l} ECM=\left(MY\times 0.327\right)+\left(FY\times 12.86\right)+\left(PY\times 7.65\right) \end{array}$$

where *MY, FY, and PY* indicate the kg of milk, fat, and protein produced within the lactation.

Milk SCC was converted to somatic cell score (SCS) according to the formula proposed by Ali and Shook (29) to achieve normal distribution:


$$\begin{array}{l} SCS\;=\;3\;+\log_{2}\left(SCC/100,000\right). \end{array}$$

Test-day SCS values were then averaged within each lactation, in order to be merged with STAY phenotypes and be used as an indicator of the cow's udder health.

#### Morphological characteristics

Linear-type traits can be scored on any days in milk (DIM) in primiparous cows. However, the morphological traits considered in the present study (muscularity and BCS) are known to vary within lactation, suggesting that observed differences among cows can be due also to the moment in which they were scored, i.e., stage of lactation. For this reason, following Buonaiuto et al. (30), individual muscularity and BCS lactation curves were adjusted through random regression analysis, allowing daily individual prediction of both traits to be present. In such a way, the differences in the expected muscularity and BCS among cows at the same DIM becomes independent from the number of days post-calving at linear type scoring. Subsequently, average lactation profiles were estimated for cows belonging to different classes of age at first calving (30), in order to evaluate the absolute growth rate (AGR) of both muscularity and BCS. The AGR was calculated according to the formula used by Handcock et al. (31):


$$\begin{array}{l} AGR\;=\;\frac{\left({BT}_{x}{-\;BT}_{y}\right)}{\left(t_{x}-t_{y}\right)} \end{array}$$

where *BT*_*x*_ and *BT*_*y*_ are the predicted muscularity or BCS at x^th^ and y^th^ DIM, *t*_*x*_ is the initial age of the cow (in days), and *t*_*y*_ is the final age (in days) (30).

**Figure 1:**
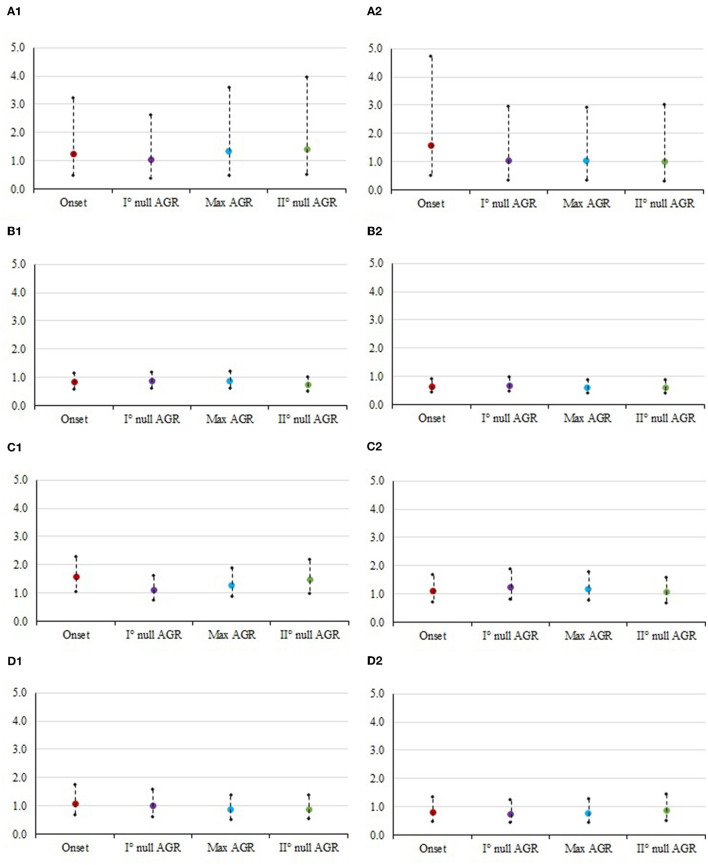
Odds ratios and 95% confidence interval for the risk of culling at each class of muscularity in different timepoints. The panels contain: (1) Mid vs. Low. **(A1)** STAY1-2, stayability as a primiparous cow, **(B1)** STAY2-3, stayability as a second-parity cow, **(C1)** STAY3-4, stayability as a third-parity cow, **(D1)** STAY4-5, stayability as a fourth-parity cow. (2) High vs. Low. **(A2)** STAY1-2, stayability as a primiparous cow, **(B2)** STAY2-3, stayability as a second-parity cow, **(C2)** STAY3-4, stayability as a third-parity cow, **(D2)** STAY4-5, stayability as a fourth-parity cow. For muscularity, timepoints were selected according to the absolute growth rates (AGR) trends reported by Buonaiuto et al. (30): onset of lactation = 5 DIM; I° null AGR = 85 DIM; Max AGR = 180 DIM; II° null AGR = 280 DIM.

For further statistical analysis, only muscularity and BCS data predicted at four specific moments during the lactation were considered (30):

i. at the onset of lactation (5 DIM for both traits; Figure 1);
ii. at the nadir of muscle and/or fat reserves losses (first null AGR), i.e., the moment where the uptake from body reserves stops in Simmental (85 and 45 DIM for muscularity and BCS, respectively);
iii. at the maximum AGR, i.e., when the greatest recovery of muscle/fat reserves is observed in Simmental (180 and 160 DIM for muscularity and BCS, respectively);
iv. at the second null AGR, representing the moment from which cows start to lose again muscle/fat reserves (280 DIM in both traits);

Subsequently, cow-specific predictions of muscularity and BCS were merged to the lactation data.

### Statistical analysis

Muscularity, BCS, ECM, and SCS (lactation average) were grouped into 5 classes based on quintile distribution for each individual variable, as: low, medium-low, medium, medium-high, and high. Records belonging to different lactations were analyzed separately, meaning that the effect of parity was not accounted for in the statistical models. Muscularity and BCS predicted at each given time point during the first lactation were included. Therefore, STAY1-2, STAY2-3, STAY3-4, and STAY4-5 were analyzed 4 times, by considering at each run classes of muscularity and BCS predicted at one out of the four different time points considered.

A logistic regression model was fitted with the GLIMMIX procedure using SAS software, version 9.4 (SAS Institute INC., Cary, NC):


$$\begin{array}{l} y_{lmnopqr}=\mu +{MU}_{l}+{BCS}_{m}+{ECM}_{n}+{CR}_{o}+{SCS}_{p}\\ +\;{Herd}_{q}+e_{lmnopqr}, \end{array}$$

where *y* is STAY1-2, STAY2-3, STAY3-4, or STAY4-5; μ is the overall intercept of the model; *MU*_*l*_ is the fixed effect of the l^th^ class (*n* = 5) of muscularity predicted at each specific aforementioned time point; *BCS*_*m*_ is the fixed effect of the m^th^ class (*n* = 5; defined according to quintiles) of BCS predicted at each specific aforementioned time point; *ECM*_*n*_ is the fixed effect of the n^th^ class (*n* = 5) of ECM; *CR*_*n*_ is the fixed effect of the n^th^ class (0 vs. 1) of conception rate at first insemination, where 1 indicates that only a single insemination is needed to achieve pregnancy and 0 otherwise; *SCS*_*o*_ is the fixed effect of the 0^th^ class (*n* = 5; defined according to quintiles) of milk SCS; *Herd*_*p*_ is the random effect of the p^th^ herd (*n* = 324) of linear classification, assumed to be distributed as $\sim N\left(0,\;\sigma_{H}^{2}\right),$ where $\sigma_{H}^{2}$ is the herd variance; and *e* is the random residual term, assumed to be distributed as $\sim N\left(0,\;\sigma_{e}^{2}\right)$ where $\sigma_{e}^{2}$ is the residual variance. For the fixed effects of muscularity and BCS the first class (low) was kept as the reference, and each odds ratio (OR) was considered significant when the 95% CI did not include 1.

## Results and discussions

### Overview of the studied population

The Italian Simmental cows included in the present research presented relatively high production levels compared to those reported by Cziszter et al. (32) for Fleckvieh (Austrian Simmental) cattle and by Erdem et al. (33) for Simmental cows reared in Turkey. The evolution of yield traits from parity 1 onwards in Italian Simmental cows are shown in Table 2. Descriptive statistics (mean ± standard deviation) indicated that the average MY in this study increased gradually from parity 1 to 3 (Table 2) and then decreased (parity 4: 6,660.04 ± 2,688.70 kg). The MY trend across parities was similar to that reported by different authors (34, 35) for different European populations of Simmental cows. Milk composition plays an important role in countries like Italy where approximately the 75% of the total national milk annually produced is used for cheese manufacturing (36–38). As a matter of fact, fat and protein content together with pH and acidity are essential factors during milk processing into finished dairy products (39).

**Table 2:** Overview of Simmental cows' productivity^a^ and fertility^b^ performance in different parities.

**Parity**	**Trait**	**Mean**	**Median**	**SD**	**Coefficient of variation, %**	**Min**.	**Max**.
1	Milk yield (kg)	6,847.58	6,702	2,152.30	31.43	149	14,754
	Fat yield (kg)	264.39	255	85.03	32.16	22	589
	Protein yield (kg)	242.59	236	76.83	31.67	6	517
	Calving age (month)	29.54	29	4.19	14.17	20	41
	IFS (n)	83.31	75	39.78	47.75	2	218
	Days open (n)	120.79	100	86.36	71.49	0	532
	Calving interval (d)	410.73	391	76.70	18.67	283	666
2	Milk yield (kg)	6,861.69	6,932	2,541.66	37.04	92	14,750
	Fat yield (kg)	265.71	262	94.81	35.68	18	589
	Protein yield (kg)	245.23	245	86.60	35.31	19	512
	Calving age (month)	43.28	42	5.37	12.41	32	65
	IFS (n)	81.17	74	38.08	46.91	6	218
	Days open (n)	109.96	92	87.54	79.61	0	858
	Calving interval (d)	401.99	382	70.17	17.46	276	663
3	Milk yield (kg)	6,947.05	7,015	2,698.66	38.85	354	14,739
	Fat yield (kg)	270.51	269	99.87	36.92	9	583
	Protein yield (kg)	247.56	247	91.72	37.05	13	517
	Calving age (month)	56.51	56	6.34	11.22	43	82
	IFS (n)	80.78	73	36.28	44.91	6	217
	Days open (n)	107.11	88	89.73	83.78	0	476
	Calving interval (d)	400.17	378	70.87	17.71	272	665
4	Milk yield (kg)	6,660.04	6,726	2,688.70	40.37	127	14,652
	Fat yield (kg)	264.56	261	97.00	36.66	3	588
	Protein yield (kg)	240.95	237	86.30	35.82	5	514
	Calving age (month)	69.23	68	7.00	10.11	53	99
	IFS (n)	80.51	72	38.25	47.51	11	218
	Days open (n)	97.62	85	83.09	85.12	0	430
	Calving interval (d)	399.19	379	68.07	17.05	301	658
5	Milk yield (kg)	6,745.17	6,827	2,920.80	43.30	355	14,489
	Fat yield (kg)	265.82	261	103.18	38.82	14	585
	Protein yield (kg)	242.58	237	94.23	38.85	12	509
	Calving age (month)	82.03	81	7.75	9.45	65	113
	IFS (n)	81.91	73	37.83	46.18	1	212
	Days open (n)	98.61	79	89.37	90.63	0	494
	Calving interval (d)	402.74	382	67.60	16.79	282	645

a Based on all test-day records.

b IFS, Interval form calving to first insemination.

Along with the high productivity, the population studied was characterized by a composition comparable to that of specialized dairy breeds, with an average fat and protein content of 3.79 and 3.48%, respectively (data not shown). Parity-specific descriptive statistics of fat and protein yield, both used to calculate ECM, are reported in Table 2. Data observed are similar to that reported by other authors (40, 41) for Simmental dairy cows. By using test-day records of the first 150 DIM, Costa et al. (42) reported an average milk, fat, and protein yield of 4,157, 167.5, and 136.4 kg, respectively in Fleckvieh cows. In the same DIM window, these authors reported fat and protein content to average 4.03 and 3.28% (42). Numerous studies have investigated the effect of parity on yield traits using both test-day records or lactation data. According to the literature (43, 44) the positive correlation between MY and parity observed until a certain parity order could be attributed to the udder development/size, i.e., to the increasing number of functional secretory cells, and to the different requirements of primiparous and multiparous.

The cows' productive level and milk quality can be evaluated simultaneously by the means of ECM. The ECM can be considered as a key parameter for STAY in dairy cows as it directly affects the farm profit and, consequently, may have an effect on culling decisions (45). The culling of unproductive cows is necessary to keep the herd profitable and is thereby done on a voluntary basis. In the field, the real objective is to reduce the involuntary culling, e.g., elimination of cows—perhaps with a good MY—due to scarce fertility, severe disease, or acute inflammation (46). Well managed herds show high survival rates, thus a great proportion of mature cows and, consequently, a lower replacement rate (47).

In the present research the highest and the lowest CI value were observed in 1st (410.73 ± 76.70 d) and 4th lactation (399.19 ± 68.07 d) and, overall, the mean CI is similar to that reported in previous research in Italian Simmental cows (30). Dry period length averaged between 75.28 ± 29.22 (parity 1) to 81.29 ± 30.65 (parity 4). Across lactation the median of dry period ranged from 72 (for parity 1) to 77 (parity 2 and 4).

An overview of the investigated STAY traits and their definition is reported in Table 1. In particular, the survival rates were 98, 70, 62, and 57% (Table 1) and of the initial 2,656 cows present in parity 1 only 642 survived until parity 5 (24%; data not shown). Results are in general difficult to be compared with the literature due to scarce information available on such traits, especially for Simmental. In Holstein, Hardie et al. (19) reported that 84% (at parity 1) and 80% (at parity 2) of Holstein cows in US organic herds were able to survive and continue their productive career. Moreover, Garcia-Peniche et al. (47), report that from 38 to 43% of Holsteins stayed until 5 years of age, completing an average of 2.12 to 2.22 lactations. In the official annual report of Zuchtdata (48) the average number of calvings is equal to 3.83 for Austrian Fleckvieh while the average productive life is estimated at 3.66 years. The 24.17% of the Italian Simmental dual-purpose cows involved in this study achieved parity 5 (Table 1) which greatly differs from what has been reported for other breeds. As an example, Hare et al. (49) reported that US Holsteins dairy cows' population experienced a serious drop in survival to parity 5: from 24.2% recorded in 1980 to 14.3% in 1998. In Ireland, Williams et al. (50) reported that only 13% of the Holsteins cows involved in their study survived to the fourth lactation. Similar results are reported by Hardie et al. (19) that observe only 14% of US organic Holstein dairy cows reach parity 5. It is worth to highlight that, for the purpose of this study, data provided by ANAPRI belong exclusively to cows that were linearly classified during the first lactation, thus with a BCS and a muscularity score available. It derives that non-linear classified scored cow (e.g., for early culling in parity 1 before scoring) are not accounted for in this study. For this reason, results of this study may be interpreted in the light of absence of data from early culled cows.

According to Padilla et al. (51), a gradual age-related body deterioration is common to most animals, including dairy cows (52) and in livestock species this can affect both health and fitness of producing animals. In the past, the selection of high-producing dairy cows has favored larger more angular females, which resulted in skinny with poor carcass yield characteristics. Differently, dual-purpose cows as Simmental are characterized by a long and muscular body, that makes back and buttocks convex in most of the cases. This different body conformation was also observed by Knob et al. (53), who reported BCS of Simmental cows (and their crosses) to be approximately 1 point higher that of Holstein cows in all stages of lactation. Differences in body conformation can partly justify the survival rate observed in dairy vs. dual-purpose breeds (18). It is important to consider that, in the case of dual purpose breeds, culling could be influenced by external and economic factors, e.g., the market demand and price of milk and meat and the feed cost. Generally, when heifers of dual-purpose breeds are abundant and meat low-priced, farmers tend to cull more than usual, increasing the herd replacement rate.

### Sources of variation of stayability

**Figure 2:**
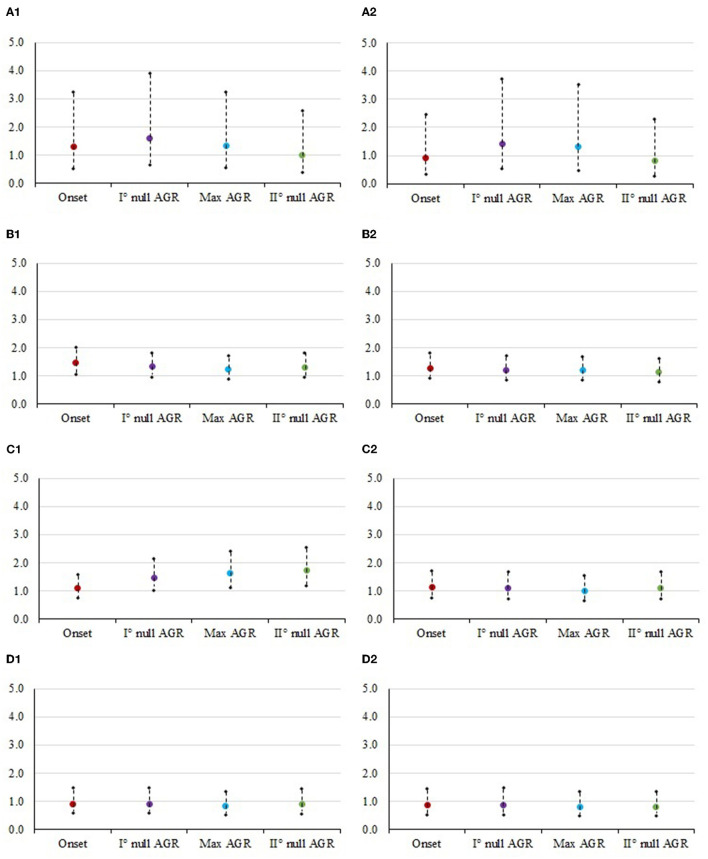
Odds ratios and 95% confidence interval for the risk of culling at each class of body condition score in different timepoints. The panels contain: (1) Mid vs. Low. **(A1)** STAY1-2, stayability as a primiparous cow, **(B1)** STAY2-3, stayability as a second-parity cow, **(C1)** STAY3-4, stayability as a third-parity cow, **(D1)** STAY4-5, stayability as a fourth-parity cow. (2) High vs. Low. **(A2)** STAY1-2, stayability as a primiparous cow, **(B2)** STAY2-3, stayability as a second-parity cow, **(C2)** STAY3-4, stayability as a third-parity cow, **(D2)** STAY4-5, stayability as a fourth-parity cow. For body condition score, timepoints were selected according to the absolute growth rates (AGR) trends reported by Buonaiuto et al. (30): onset of lactatio*n* = 5 DIM; I° null AGR = 45 DIM; Max AGR = 160 DIM; II° null AGR = 280 DIM.

Results from the analysis of variance for STAY traits are summarized in Table 3. In the case of muscularity and BCS effect, the odds ratio estimates are depicted in Figures 1, 2, whereas estimates obtained for levels of SCS and CR are presented in Table 4. Overall, the effect of ECM, CR, and SCS were always significant indicating that some odds ratios differed (*P* < 0.001; Table 4), with the only exception of STAY1-2. Apart from severe reasons, in fact, Italian Simmental cows, whose average productive life is 3.3 lactations, are generally kept in the herd at least until second calving, i.e., regardless of the performance (26). This may partly explain why STAY1-2 was not affected by the above-mentioned fixed effects. Inclusion of ECM, CR and SCS allowed to account for that variability related to productivity level, udder health, and fertility. Odds ratio of these effects (Table 4) generally indicate that STAY is associated with SCS levels. In fact, as SCS increases, the risk of culling also increased; similar trends were observed for ECM. Regarding CR, we observed a higher risk of culling in cows with low CR (Table 4). However, although the odds ratio showed an association for all these effects, the significances was always >0.05.

**Table 3:** F-values and significance of fixed effects^a^ included in the analysis of stayability traits.

**Trait**	**Moment^b^**	**MU**	**BCS**	**ECM**	**CR**	**SCS**	**Herd variance**	**RSD**
STAY1-2	Onset	3.00^*^	0.73	1.39	2.03	1.26	0.08	0.30
	I° null AGR	1.28	0.61	1.36	1.99	1.16	0.09	0.33
	Max AGR	1.59	0.58	1.32	2.13	1.21	0.08	0.31
	II° null AGR	0.77	1.44	1.32	1.88	0.97	3.39	3.16
STAY2-3	Onset	2.12	3.69^*^	47.98^***^	97.59^***^	5.05^**^	0.28	0.10
	I° null AGR	1.45	3.86^**^	47.92^***^	98.36^***^	5.04^**^	0.28	0.10
	Max AGR	2.04	2.69^*^	47.75^***^	98.24^***^	5.06^**^	0.28	0.10
	II° null AGR	1.87	2.95^*^	47.51^***^	95.98^***^	4.77^**^	0.29	0.10
STAY3-4	Onset	0.27	1.90	34.31^***^	74.07^***^	5.22^**^	0.45	0.14
	I° null AGR	0.40	1.19	34.15^***^	75.05^***^	5.07^**^	0.44	0.14
	Max AGR	1.00	2.84^*^	34.77^***^	75.08^***^	5.22^**^	0.45	0.14
	II° null AGR	2.72^*^	2.50^*^	34.74^***^	76.29^***^	5.22^**^	0.43	0.14
STAY4-5	Onset	0.61	0.52	24.47^***^	56.35^***^	3.25^*^	0.23	0.15
	I° null AGR	0.60	0.37	24.42^***^	56.48^***^	3.29^*^	0.22	0.14
	Max AGR	0.39	0.45	24.47^***^	55.55^***^	3.47^**^	0.21	0.14
	II° null AGR	0.43	0.79	24.72^***^	55.77^***^	3.67^**^	0.23	0.14

* *p* < 0.05;

** *p* < 0.01;

*** *p* < 0.001.

a MU, muscularity; BCS, body condition score; ECM, energy corrected milk; CR, conception rate; SCS, somatic cell score; RSD, residual standard deviation.

b For either the MU or BCS effect. Based on absolute growth rates (AGR) trends reported by Buonaiuto et al. (30): onset of lactation = 5 days in milk; I° null AGR = 85 days in milk for MU and 45 for BCS; Max AGR = 180 days in milk for MU and 160 for BCS; II° null AGR = 280 days in milk.

**Table 4:** Odds ratios (95% confidence interval) for the risk of culling at each class of energy corrected milk, somatic cell score, or conception rate estimated from the different time points^a^.

**Trait**	**Moment^a^**	**Energy corrected milk** ^**b**^		**Conception rate^c^**	**Somatic cell score** ^**b**^	
		**Medium**	**High**	**0**	**Medium**	**High**
STAY1-2	Onset	0.55 (0.21–1.42)	0.36 (0.14–0.93)	1.54 (0.85–2.78)	1.61 (0.68–3.83)	1.11 (0.52–2.40)
	I° null AGR	0.55 (0.21–1.42)	0.37 (0.15–0.95)	1.53 (0.85–2.77)	1.61 (0.68–3.84)	1.13 (0.53–2.44)
	Max AGR	0.55 (0.21–1.43)	0.37 (0.15–0.96)	1.55 (0.86–2.80)	1.66 (0.70–3.96)	1.13 (0.53–2.44)
	II° null AGR	0.45 (0.16–1.29)	0.33 (0.11–0.94)	1.56 (0.83–2.92)	1.57 (0.62–3.99)	1.02 (0.44–2.37)
STAY2-3	Onset	5.34 (3.91–7.29)	4.60 (3.40–6.23)	0.35 (0.28–0.43)	1.29 (0.95–1.74)	1.01 (0.76–1.36)
	I° null AGR	5.33 (3.90–7.27)	4.62 (3.41–6.26)	0.35 (0.28–0.43)	1.28 (0.95–1.72)	1.01 (0.75–1.35)
	Max AGR	5.27 (3.86–7.19)	4.61 (3.41–6.24)	0.35 (0.29–0.43)	1.30 (0.96–1.75)	1.00 (0.75–1.35)
	II° null AGR	5.28 (3.87–7.21)	4.60 (3.40–6.23)	0.36 (0.29–0.44)	1.28 (0.95–1.72)	1.00 (0.75–1.34)
STAY3-4	Onset	5.65 (3.90–8.16)	6.26 (4.29–9.14)	0.35 (0.28–0.45)	1.64 (1.16–2.32)	1.21 (0.86–1.71)
	I° null AGR	5.64 (3.90–8.15)	6.18 (4.24–9.02)	0.35 (0.28–0.45)	1.62 (1.15–2.29)	1.22 (0.86–1.72)
	Max AGR	5.79 (4.00–8.39)	6.39 (4.37–9.34)	0.35 (0.28–0.45)	1.64 (1.16–2.32)	1.24 (0.88–1.75)
	II° null AGR	5.71 (3.94–8.25)	6.30 (4.31–9.20)	0.35 (0.27–0.44)	1.63 (1.15–2.31)	1.24 (0.88–1.75)
STAY4-5	Onset	6.11 (3.86–9.66)	7.32 (4.53–11.83)	0.32 (0.24–0.43)	1.80 (1.17–2.78)	1.23 (0.80–1.88)
	I° null AGR	6.03 (3.82–9.52)	7.23 (4.74–11.67)	0.32 (0.24–0.43)	1.83 (1.19–2.82)	1.23 (0.80–1.87)
	Max AGR	5.96 (3.78–9.39)	7.22 (4.48–11.64)	0.32 (0.24–0.44)	1.86 (1.21–2.86)	1.23 (0.80–1.87)
	II° null AGR	6.03 (3.82–9.51)	7.21 (4.47–11.61)	0.32 (0.24–0.43)	1.90 (1.23–2.94)	1.25 (0.82–1.91)

a For either the muscularity (MU) or body condition score (BCS) effect. Based on absolute growth rates (AGR) trends reported by Buonaiuto et al. (30): onset of lactation = 5 days in milk; I° null AGR = 85 days in milk for MU and 45 for BCS; Max AGR = 180 days in milk for MU and 160 for BCS; II° null AGR = 280 days in milk.

b “Low” is the reference class.

c CR = 1 (reference class) indicates that only a single insemination was needed to achieve pregnancy, otherwise CR = 0.

Muscularity was significant for STAY1-2 at the onset of lactation and for STAY3-4 in second null AGR and in late lactation. On the other hand, BCS was significant during all the phases considered for STAY2-3 (*P* < 0.05). STAY3-4 was significantly (*P* < 0.05) affected by cow's BCS during the max (around 160 DIM), and the second null AGR (around 280 DIM).

Figures 1, 2 show the odds ratio of the risk of culling from STAY1-2 to STAY 4-5 in cows showing different muscularity and BCS level. Although not significant in most of the cases (Table 3), the odds ratio generally indicates that animals with an average condition in terms of both muscularity and BCS are exposed to a lower risk of culling compared to cows with lower (sub-optimal) scores.

In particular, Italian Simmental dual-purpose cows with medium BCS at the beginning of lactation are more likely (1.3 times greater in the case of STAY1-2) to complete the lactation compared to those with lower condition (Figure 2A). From the moment of greatest recover of muscle and fat reserves (approximately at 180 DIM) to the moment after which animals lose again muscle and fat tissue (approximately at 280 DIM), dairy cows with medium conditions are more likely to stay in the herd, compared to cows with low condition (Figures 1, 2). An example is given by the odds ratios of STAY2-3 which are depicted in Figure 1B; in fact, the cows' muscularity had a strong impact on productive life, and therefore in the ability to stay in the herd. Indeed, at STAY2-3, cows whose muscularity was classified as high are less likely to stay in the herd compared to cows with a low muscularity, with odds ratio at DIM 5, 85, 180, and 280 being lower than unity and equal to 0.639, 0.690, 0.619, and 0.612. Conversely, at STAY2-3, cows with high BCS (Figure 2A) are more likely to stay in herd compared to cows with a low BCS, especially at 45 (odds ratio = 1.404) and 180 DIM (odds ratio = 1.310). The odds ratios (Figure 2C) show how, in parity 3, the BCS has a strong and significant impact on the cows' STAY (STAY3-4), with cows in the medium class being more likely to stay in the herd compared to those in the low class; the odds ratio at 45, 160, and 280 DIM was 1.470, 1.639, and 1.724, respectively. The same can be valid for cows with high BCS compared to those with low BCS (5 DIM: 1.129, 45 DIM: 1.102, 160 DIM: 1.005, 280 DIM: 1.100). During STAY3-4 cows with a medium muscularity condition are more likely to stay in the herd compared to cows with low muscularity, presenting an odds ratio of 1.569 at 5 DIM. At 280 DIM, the moment after which animals lose again muscle tissue, cows with medium BCS are significantly more likely (odds ratio = 1.724) to continue their career compared to those whose condition was classified as low. Similar result could be observed at STAY4-5 (Figure 1D1), in particular, cows with medium muscularity at onset of lactation (5 DIM) are more likely to stay in herd compared to cows with low conditions (odds ratio: 1.091). In general, the result depicts a fall in the probability to stay in the herd for cows with high muscularity and BCS conditions (Figures 1D2, 2D2). For example, cows with high muscularity conditions are less likely to stay in herd compared to cows with low conditions (odds ratio: 5 DIM: 0.806, 45 DIM: 0.784, 160 DIM: 0.775, 280 DIM: 0.872, Figure 1D2).

Potential reasons that can explain some of the results observed may be related to the status of negative energy balance (NEB) that commonly occurs in the periparturient period (54). Grummer et al. (55) estimated energy balance to be around−5.8 Mcal/d when cows are close to parturition, with peaks up to−20 Mcal/d during the 1st month of lactation. Plaizier et al. (56) reported that, in addition to NEB, cows can also experience a negative nitrogen balance in the 1st days after calving. During this phase, dairy cows, especially high-producing ones, cannot fulfill the energy deficit by increasing their feed intake (57). According to what has been reported by Straczek et al. (58), lactating dairy cows are characterized by high plasma levels of leptin, an anorectic hormone, directly related to a high loss of body condition caused by intensive lactogenesis. Therefore, cows are forced to mobilize body reserves, like fat and muscle tissue (33, 58–60). Indeed, even if body fat tissue is identified as the major body source of energy reserves, the catabolism of protein may also contribute to nutrient requirements especially in primiparous and/or early lactation (61). According to Komaragiri et al. (62), during early lactation a cow can lose around 20 kg of muscular tissue and between 8 to 57 kg of body fat. In particular, van der Drift et al. (54) found out that fat mobilization that starts immediately after calving continues up to the 8^th^ week after parturition. Also Schäff et al. (63) observed that skeletal muscle mobilization takes place, starting immediately after calving, but the duration was shorter. In fact, it stopped at about 5 weeks postpartum, with a peak mobilization rate during the first 2 weeks of lactation (63). Findings by Megahed et al. (60) are in accordance with our results, particularly with the fact that body condition of cows facing up to the second calving is crucial to deciding their survival in the herd. Indeed, Megahed et al. (60) reported a greater periparturient mobilization of backfat and skeletal muscle in primiparous than in multiparous cows. The reasons for such greater mobilization in younger animals could be related to the reason they have not finished their growth yet (64) and that they have to cope also with growing requirements, in addition to production and maintenance. Straczek et al. (58) reported that Simmental cows have a greater capacity to adjust the NEB state compared to Holsteins, restoring earlier the BCS loss after the lactation peak. Consequently, cows with good conditions at the onset of second lactation are more prone to perform better along the lactation and to be more resilient to the different metabolic disorders and reduced fertility (65). It is worth considering that farmers rearing Simmentals may decide to cull cows with higher muscularity at a certain point for beef purposes in order to increase the herd profit. Although cows with high BCS are more likely to stay in herd compared to those with low BCS (Figure 2B), the odds ratios for cows with medium BCS were always the highest. Similar results were observed by Erdem et al. (33), who suggested that rearing cows with moderate BCS conditions can be considered an important approach for the herd management. This implies that farmers prefer to cull fat cows to leave space for animals with a medium condition. Probably, dairy farmers are interested to rear cows with appropriate BCS (around 3.0 on a 5-point scale) because these parameter plays an important role in maintaining the health status of lactating cows. As reported by Yasothai (66), dairy cows presenting a severe BCS loss during lactation are exposed to several reproductive problems resulting in longer intervals between first ovulation and estrus, more days open, and lower first-service conception rates. Moreover, literature demonstrates that dairy cows with BCS greater than 3.5 tend to exhibit several metabolic disorders, such as hypocalcaemia, fatty liver, oxidative stress and ketosis (67–70). In addition, fat or over-conditioned dairy cows are at higher risk of developing a combination of metabolic, digestive, infectious and reproductive conditions known as the “fat cow syndrome” (71, 72). Bahrami-Yekdangi et al. (73) reported that in over-conditioned cows (BCS > 3.75; odds ratio = 1.27) the incidence of dystocia was larger than in other cows. An excessive accumulation of body fat predisposes to more insulin resistance, especially during the prepartum, a metabolic disorder with characteristics similar to human type 2 diabetes (74, 75). A transitory phase state of insulin resistance is generally considered a homeorhetic adaptation during early lactation, which provides glucose supply to the mammary gland limiting glucose utilization by insulin-responsive peripheral tissues, such as skeletal muscle or adipose tissue (76). Furthermore, insulin resistance can increase lipolysis of adipose tissue, and the accumulation of non-esterified fatty acids leads in turn to increased insulin resistance. In addition, the high culling risk generally observed for fat cows could be related to the negative relationship between high BCS and milk production (77).

## Conclusions

In the present study data of Italian Simmental cows were used to investigated the relationship between STAY and type traits, namely muscularity and BCS. The results indicate that cows characterized by a medium BCS/muscularity are more likely to stay in the herd compared to those with extreme body conditions, i.e., they are more likely to close the lactation and then start the subsequent one. Results of this study provide new insights into the survival and culling of Italian Simmental cattle population. Apart from productivity, in dual-purpose cows type traits and STAY are connected, being indicators of direct voluntary culling with a direct effect on farm's profitability. Further studies should disclose genetic architecture of STAY taking into account muscularity, BCS, and productive performance.

## Data availability statement

The raw data supporting the conclusions of this article will be made available by the authors, without undue reservation.

## Ethics statement

This study did not require manipulation or modification of the usual handling of the animals, since we have worked directly with the routine records provided by the breeders' associations.

## Author contributions

Conceptualization: AF, LD, and GV. Methodology: GV and LD. Software: AC and GV. Formal analysis and writing—original draft preparation: GB, GV, and AC. Data curation: LD, AC, GB, and GV. Writing—review and editing: GV, GN, AC, and NL-V. Visualization: AF, DC, AP, and LM. Supervision: GV, AF, and NL-V. Project administration: GV and AF. All authors have read and agreed to the published version of the manuscript.
